# CDK7 is a component of the integrated stress response regulating SNAT2 (SLC38A2)/System A adaptation in response to cellular amino acid deprivation

**DOI:** 10.1016/j.bbamcr.2019.03.002

**Published:** 2019-06

**Authors:** Clare Stretton, Christopher Lipina, Russell Hyde, Emma Cwiklinski, Thorsten M. Hoffmann, Peter M. Taylor, Harinder S. Hundal

**Affiliations:** Division of Cell Signalling and Immunology, Sir James Black Centre, School of Life Sciences, University of Dundee, Dundee DD1 5EH, UK

**Keywords:** GSK3, Roscovitine, Transporter, Me-AIB, GCN2, ATF4

## Abstract

Extracellular amino acid (AA) withdrawal/restriction invokes an integrated stress response (ISR) that induces global suppression of protein synthesis whilst allowing transcription and translation of a select group of genes, whose protein products facilitate cellular adaptation to AA insufficiency. Transcriptional induction of the System A/SNAT2 AA transporter represents a classic adaptation response and crucially depends upon activation of the General Control Nonderepressible-2 kinase/Activating transcription factor 4 (GCN2/ATF4) pathway. However, the ISR may also include additional signalling inputs operating in conjunction or independently of GCN2/ATF4 to upregulate SNAT2. Herein, we show that whilst pharmacological inhibition of MEK-ERK, mTORC1 and p38 MAP kinase signalling has no detectable effect on System A upregulation, inhibitors targeting GSK3 (*e.g.* SB415286) caused significant repression of the SNAT2 adaptation response. Strikingly, the effects of SB415286 persist in cells in which GSK3α/β have been stably silenced indicating an off-target effect. We show that SB415286 can also inhibit cyclin-dependent kinases (CDK) and that roscovitine and flavopiridol (two pan CDK inhibitors) are effective repressors of the SNAT2 adaptive response. In particular, our work reveals that CDK7 activity is upregulated in AA-deprived cells in a GCN-2-dependent manner and that a potent and selective CDK7 inhibitor, THZ-1, not only attenuates the increase in ATF4 expression but blocks System A adaptation. Importantly, the inhibitory effects of THZ-1 on System A adaptation are mitigated in cells expressing a doxycycline-inducible drug-resistant form of CDK7. Our data identify CDK7 as a novel component of the ISR regulating System A adaptation in response to AA insufficiency.

## Highlights

• Roscovitine and flavopiridol (CDK inhibitors) block the System A adaptive response.
• Extracellular amino acid (AA) withdrawal induces CDK7 activation.
• Pharmacological inhibition of GCN2 represses CDK7 activation in AA-deprived cells.
• Targeted suppression of CDK7 represses ATF4 expression and System A adaptation.

## 1 Introduction

The solute carrier 38 (SLC38) transporter family comprises six functionally characterised amino acid (AA) transporters from the eleven members that have been identified in the human genome and notably include two sub groups whose activities align with those classically described as System A or System N [9]. The System A transporters include SNAT1, SNAT2 and SNAT4 (*aka* SLC38A1, SLC38A2 and SLC38A4, respectively) and these mediate the sodium-dependent uptake of short chain neutral AAs such as alanine, serine and threonine. System A was functionally characterised by its ability to accept N-alkylated substrates such as α-(methyl-amino)isobutyric acid (MeAIB), whereas, those of the System N family, which include SNAT3, SNAT5 and SNAT7 (*aka* SLC38A3, SLC38A5 and SLC38A7 respectively), do not accept Me-AIB but show preference for AAs containing an extra nitrogen in their side chains (*i.e.* glutamine, asparagine and histidine) as substrates and, moreover, exhibit tolerance for lithium as a sodium substitute [26].

Whilst transporters of the System A sub group share significant sequence homology, it is widely established that SNAT2 (SLC38A2) is the most ubiquitously expressed and, strikingly, one of the most extensively regulated AA transporters to have been documented to date, possibly reflecting its important contribution to cellular AA nutrition and to the control of diverse cellular functions. SNAT2 expression/activity is, for example, subject to both acute and chronic modulation by hormones (*e.g.* glucocorticoids, estrogen, insulin) and growth factors [2,20,24,55]. In tissues, such as the mammary gland, the transcriptional upregulation of SNAT2 by 17β-estradiol may play a significant role in meeting the increased AA demand that facilitates differentiation and proliferation of this tissue in preparation for lactation [55], whereas, in skeletal muscle, recruitment of SNAT2 carriers from an intracellular compartment to the plasma membrane and the attendant increase in AA delivery in response to insulin may form part of the anabolic effect that the hormone has upon muscle protein synthesis [20,24]. SNAT2 can also be upregulated in cells subjected to hyperosmotic stress; a response designed to elevate cellular intake of organic osmolytes (AAs) that helps establish an osmotic drive for water uptake into cells to restore both intracellular volume and ionic strength [6,10,36]. Crucially, the sodium coupled uptake of extracellular AAs establishes an outwardly-directed concentration gradient of SNAT substrates, which, if not immediately utilised for metabolic processes, can leave the cell *via* tertiary exchange transporters, such as the leucine-preferring (LAT1) carrier, that operates in parallel with SNAT2 in the plasma membrane [5,21]. This SNAT2/LAT1 exchange coupling is considered significant for intracellular leucine delivery given that this essential AA serves to potently activate the mTORC1/S6K1 signalling axis [33]. The mechanistic target of rapamycin complex 1 (mTORC1) plays a pivotal role in the control of mRNA translation, cell growth/metabolism and autophagy [50] and consequently factors affecting SNAT2 expression/activity will indirectly impact on the regulation of these key cellular processes by virtue of the changes that occur in mTORC1 activity [47,54].

Whilst AA insufficiency, even of a single AA such as methionine or leucine, exerts a profound suppressive effect on global mRNA translation [37], the expression and translation of a sub-set of genes that allow cellular adaptation to changes in environmental nutrient supply is upregulated [25]. A key mediator of this amino acid response (AAR) is the general control nonderepressible-2 kinase (GCN2), which, in response to AA insufficiency, is activated by the binding of uncharged tRNAs that result in eIF2α phosphorylation and a reduction in global mRNA translation [11]. Paradoxically, however, the translation of specific mammalian mRNA transcripts such as those encoding activating transcription factor 4 (ATF4), or enzymes involved in AA biosynthesis (*e.g.* asparagine synthase), AA transport (*e.g.* SNAT2) and that of the ATF4-dependent transcription factor CHOP (C/EBP Homologous Protein) is elevated [27,35]. The transcriptional increase in SNAT2 expression is dependent upon an AA responsive domain in the first intron of the *SLC38A2* gene activated by ATF4 [43]. However, for some of these targets, such as SNAT2, the adaptive increase in expression/function is also accompanied by enhanced protein stabilization *via* a process that involves its reduced ubiquitination and degradation [23]. Moreover, in the case of SNAT2, the extracellular presence and/or binding of sodium also appears to be a requisite in supporting the genomic increase in SNAT2 expression and its enhanced protein stability in response to AA insufficiency [19]. This latter observation implies that, in addition to the GCN2/ATF4 pathway, additional mechanisms are likely to contribute to the integrated stress adaptation response. Indeed, the notion that additional signalling inputs may form part of the AAR is supported by data showing that in HEK293T and HepG2 cells the upregulation of the early growth response-1 gene induced by either AA deprivation or endoplasmic reticulum (ER) stress is independent of the GCN2/ATF4 pathway and instead involves activation of MEK-ERK signalling [51]. ER stress has also been shown to promote induction of SNAT2 [42] and CHOP and, at least in the case of the latter, this induction is mediated *via* an ATF-4 independent mechanism that can be repressed by inhibitors of Glycogen Synthase Kinase-3 (GSK3) [38]. Precisely how GSK3 regulates ER-induced CHOP expression is currently unclear, and whether it or MEK-ERK signalling form part of the AAR underpinning adaptive changes in SNAT2 expression/activity remains currently unknown.

In the current investigation we show that whilst inhibition of MEK-ERK, mTORC1 and p38 MAP kinase signalling have no impact on the adaptive increase in SNAT2 activity induced by AA insufficiency, two structurally unrelated GSK3 inhibitors significantly blunt the SNAT2 response to AA withdrawal. Intriguingly, however, the repressive action of GSK3 inhibitors on SNAT2 adaptation persists in cells in which GSK3α/β are substantially depleted by shRNA indicating that the pharmacological effects of these inhibitors most likely represent an off-target effect. Our analysis reveals that this off-target effect is most likely mediated *via* cyclin-dependent kinases (CDK) and in particular CDK7, which we identify as a novel AA regulated kinase. Here we demonstrate that a potent and selective inhibitor of CDK7 attenuates the SNAT2 adaptation response and that this repressive action can be mitigated in cells expressing a doxycycline-inducible drug-resistant form of CDK7.

## 2 Materials and methods

### 2.1 Reagents

α-MEM (α-minimal essential medium), DMEM (Dulbecco's modified Eagle's medium), OPTI-MEM, Lipofectamine 2000 and antibiotic/antimycotic solution were from Invitrogen (Carlsbad, CA). EBSS was obtained from Sigma-Aldrich (St Louis, MO). FBS was purchased from BioSera (France). Restriction enzymes and other DNA-modifying enzymes were purchased from either New England Biolabs (Beverley, MA) or Roche Diagnostics (Burgess Hill, UK). Olignucleotides were purchased from the University of Dundee Oligo Synthesis Service (University of Dundee). Anti-Slc38a2 (SNAT2) antibody was from MBL, anti-α-subunit Na/K-ATPase antibody was from the Developmental Studies Hybridoma Bank (University of Iowa), anti-β actin, anti-GAPDH and anti-Flag antibodies were from Sigma-Aldrich, anti-V5 and Alexa-Fluor488 anti-mouse antibodies were from Invitrogen. Antibodies against GSK3α/β, CDK5, p35, CDK7 and CDK9 were purchased from Santa Cruz Biotechnology (Dallas, TX). HRP-conjugated anti-mouse and anti-rabbit secondary antibodies were purchased from Cell Signaling Technologies (Beverley, MA). THZ-1 was from MedChemExpress (Sweden) and BS-181 was purchased from Cayman Chemical (Ann Arbor, MI). THZ-531 was a kind gift from Dr. Nathaneal Gray (Dana-Farber Cancer Institute, Harvard Medical School). Go Taq DNA polymerase, deoxyribonucleotide phosphates (dNTPs) and Moloney Murine Leukemia Virus (M-MLV) reverse transcriptase were purchased from Promega (Madison, WI). [^32^P]-ATP, [^14^C]-MeAIB and protein G-sepharose were from GE Healthcare (Chicago, IL). Cycloheximide, ATF4 and tubulin antibodies were purchased from Abcam (Cambridge, UK). PD098059, SB415286, SB216763 and rapamycin were purchased from Tocris Biosciences (Abingdon, UK). Roscovitine and flavopiridol were purchased from Santa Cruz Biotechnology. All other reagents were purchased from Sigma-Aldrich or VWR unless stated otherwise.

### 2.2 Cell culture

L6 rat skeletal muscle cells were grown as described previously in α-MEM containing 2% (v/v) FBS [18]. Experiments were carried out at the myotube stage, typically 7 days post-seeding. The generation of a stable GSK3α/β double knockdown L6 cell line and the pC3luc L6 cell line have been described previously [23,53]. HeLa S3 cells stably expressing the doxycycline-inducible wild type or drug-resistant Flag-CDK7 C312S construct or the empty vector [29] were kindly provided by Dr. Nathanael Gray (Harvard Medical School, Boston). Hela S3 cells were plated in DMEM/ 10% FBS/ 1% p/s medium supplemented with 1 mg/ml G418 and 2 μg/ml puromycin. When cells were 80% confluent, 2 μg/ml doxycycline was added for 24 h. Cells were used for AA uptake studies or harvested and lysates probed with an anti-C-terminal CDK7 (SCBT sc-365075) and anti-FLAG antibodies. Human embryonic kidney (HEK) 293T cells, Ad293 cells and HeLa cells were maintained in DMEM containing 10% (v/v) FBS. Amino acid deprivation was carried out by cell incubation in EBSS. Amino acid resupply was carried out by incubation in EBSS containing a complete mix of amino acids at plasma physiological concentrations. Pharmacological compounds were added as described in the text and figure legends.

### 2.3 SDS-PAGE and immunoblotting

Cells were incubated and treated as described in the text and figure legends. After treatment cells were washed twice in ice-cold PBS and then lysed as described previously or used for preparation of total cell membranes [18]. Protein concentrations in final lysates and membrane preparations were determined using the Bradford method [7]. Cell lysates and membrane preparations were separated using SDS-PAGE, transferred onto PVDF membrane (Millipore) and immunoblotted with the appropriate antibodies as described in the text and figure legends followed by incubation with the appropriate horseradish peroxidase (HRP)-conjugated anti IgG antibody. Proteins were detected using ECL exposure to autoradiographic film (Konica Minolta (Tokyo, Japan)). Quantification of immunoblots was carried out using Image J software (NIH).

### 2.4 Cloning into adenoviral expression vector and generation of adenoviral particles

Adenoviral constructs were generated using the AdEasy Adenoviral Vector System (Agilent). Rat CDK5, CDK7, CDK9 and p35 sequences were cloned into pShuttleCMV at the *Not*I and either *Xho*I or *Eco*RV restriction sites using the oligos described in Table 1. A C-terminal V5 tag was introduced for the CDKs and a C-terminal FLAG tag for p35. Site-directed mutagenesis to generate dominant negative (DN) mutants was carried out using the QuikChange method and the oligos described in Table 1. Plasmids were either transfected directly into HEK293T cells as described below or used to generate adenoviral vectors as follows. Plasmids were digested with *Pme*I and purified using phenol/chloroform extraction followed by isopropanol precipitation. 50 μg linearised vector was transformed into BJ5183 *E. coli* cells that had previously been transformed with pAdEasy vector and rendered competent again by treatment with calcium chloride. Plasmids were prepared from smaller recombinant colonies and transformed back into *E. coli* XL-Blue cells and plasmids prepared again. Recombination was confirmed by restriction digest analysis with *Pac*I. To generate adenovirus particles pShuttleCMV vectors were digested with PacI and purified using the phenol/chloroform method and isopropanol precipitation. 4 μg digested plasmid was transfected into a 25 cm^2^ flask of Ad293 using 20 μl Lipofectamine 2000 in OPTI-MEM. Transfections were carried out for 4 h at 37 °C then the culture medium replaced with complete medium. Cells were incubated for 7 days then washed in PBS and scraped into 1 ml PBS. Cell suspensions were freeze-thawed 4 times then centrifuged at 1000 rpm for 5 min. The supernatant was collected and used to infect a fresh 25cm^2^ flask of Ad293. Adenovirus was harvested when approximately 50% of the cells were detached from the flask surface (7–10 days) by scraping and freeze-thawing as before. High-titre viral stocks were generated by repeating the harvesting/infection protocol twice more. The amount of virus needed for transductions was determined using immunofluorescence as follows. L6 cells were seeded onto glass coverslips in multi-well plates and transduced with increasing amounts of adenovirus preparation at 3 days post-seeding by washing in serum-free medium and incubating with the virus in a minimal volume of medium for 2 h before replacing the medium with complete medium. Cells were fixed in 4% paraformaldehyde at the myotube stage and immunofluorescence carried out as described previously [53] using either V5 or FLAG antibodies at the concentrations recommended by the manufacturer and anti-mouse Alexa-Fluor488 secondary antibody. Fluorescence microscopy was used to determine the dose of virus required to ensure that >90% cells were expressing the recombinant protein. L6 cells used in experiments were transduced in a similar manner as those for immunofluorescence using the dose of virus found to transduce >90% of the cells.

**Table 1:** Oligos used for cloning (A), real-time PCR (B) and generation of shRNA lentiviral constructs (C).

(A) Cloning oligos	
Oligo	Sequence (5′-3′) Top line: Forward; Bottom line: Reverse
Cloning	
CDK5	ATCGACGCGGCCGCGCCACCATGCAGAAATACGAGAAACTG
	ATCGACCTCGAGCTACGTAGAATCGAGACCGAGGAGAGGGTTAGGGATAGGCTTACCCGGGGGACAGAAGTCAGAG
P35	ATCGACGCGGCCGCGCCACCATGGGCACGGTGCTGTCCCTG
	ATCGACCTCGAGCTACTTGTCATCGTCGTCCTTGTAGTCCCGATCGAGCCCCAGGAG
CDK7	ATCGACGCGGCCGCGCCACCATGGCTGTGGACGTGAAATC
	ATCGACGATATCCTACGTAGAATCGAGACCGAGGAGAGGGTTAGGGATAGGCTTACCAAAAATTAGCTTCTTGGGC
CDK9	ATCGACGCGGCCGCGCCACCATGGCCAAGCAGTACGACTC
	ATCGACGATATCCTACGTAGAATCGAGACCGAGGAGAGGGTTAGGGATAGGCTTACCGAAGACACGTTCAAATTC
Site-directed mutagenesis	
CDK5 D144N	GAACTGAAATTGGCTAATTTTGGCCTGGCCCGA
	TCGGGCCAGGCCAAAATTAGCCAATTTCAGTTC
CDK7 D155A	TTCTGAAACTGGCAGCTTTTGGCCTGGCCAA
	TTGGCCAGGCCAAAAGCTGCCAGTTTCAGAA
CDK9 D167A	TCCTAAAGCTGGCAGCTTTTGGGCTGGCTCG
	CGAGCCAGCCCAAAAGCTGCCAGCTTTAGGA


(B) qPCR oligos	
Oligo	Sequence (5′-3′) Top line: Forward; Bottom line: Reverse
SNAT2 (rat)	ACCCTCACTGTCCCCGTCGTC
	AAGGCCAGGATCGTCACAGTAATG
SNAT2 (human)	GTGTTAATGGCTGTGACCCTGAC
	GAGACTATGACGCCACCAACTGA
CDK5 (rat)	TGGGGAAGGCACCTACGGAACT
	GAGGGCTGAACTTGGCACACC
CHOP (rat)	ACGTCGATCATACCATGTTGAAG
	GGTTTCTGCTTTCAGGTGTGG
CHOP (human)	AATGGGGGTACCTATGTTTCAC
	TCAGTCAGCCAAGCCAGAGA
ATF3 (human)	AGACGGAGTGCCTGCAGAAAGAGT
	GTGGGCCGATGAAGGTTGAGC
EGR1 (human)	CTGACCGCAGAGTCTTTTCCT
	GCCACTGACCAAGCTGAAGA
ATF4 (human)	AACCGACAAAGACACCTTCG
	ACCCATGAGGTTTGAAGTGC
GAPDH (rat/human)	TGGAAAGCTGTGGCGTGAT
	GCTTCACCACCTTCTTGAT


(C) shRNA oligos	
Oligo	Sequence (5′-3′) Top line: Forward; Bottom line: Reverse
shRNA 148	CCGG**CGGGAGATCTGTCTACTCAAA***CTCGAG***TTTGAGTAGACAGATCTCCCG**TTTTTG
	AATTCAAAAA**CGGGAGATCTGTCTACTCAAA***CTCGAG***TTTGAGTAGACAGATCTCCCG**
shRNA 298	CCGG**CCTGAGATTGTGAAGTCACTC***CTCGAG***GAGTGACTTCACAATCTCAGG**TTTTTG
	AATTCAAAAA**CCTGAGATTGTGAAGTCACTC***CTCGAG***GAGTGACTTCACAATCTCAGG**
shRNA 529	CCGG**AAGCTGTACTCCACGTCCATC***CTCGAG***GATGGACGTGGAGTACAGCTT**TTTTTG
	AATTCAAAAA**AAGCTGTACTCCACGTCCATC***CTCGAG***GATGGACGTGGAGTACAGCTT**
shRNA 723	CCGG**GTACCCAGCTACAACATCCTT***CTCGAG***AAGGATGTTGTAGCTGGGTAC**TTTTTG
	AATTCAAAAA**GTACCCAGCTACAACATCCTT***CTCGAG***AAGGATGTTGTAGCTGGGTAC**
shRNA 795	CCGG**CCTGTTGAAGTGTAACCCAGT***CTCGAG***ACTGGGTTACACTTCAACAGG**TTTTTG
	AATTCAAAAA**CCTGTTGAAGTGTAACCCAGT***CTCGAG***ACTGGGTTACACTTCAACAGG**

For (C) each hairpin consisted of a 21-nucleotide sense sequence, a short hairpin sequence (CTCGAG), a 21-nucleotide antisense sequence, and five thymidines (a stop signal for RNA polymerase). Sense and antisense strands are in bold, hairpin loop is indicated by italics.

### 2.5 Amino acid uptake assay

Methyl-aminoisobutyric (MeAIB) acid uptake assays were performed as described previously [18,23]. L6 myotubes were seeded in 12 well plates and transduced at 3 days post-seeding with an amount of virus found to lead to expression of the relevant protein in over 90% of the cells as determined in the methods above. MeAIB uptake assays were carried out when the cells reached the myotube stage (approximately 7–8 days post-seeding). HEK293T cells were transfected with the relevant plasmids the day after seeding. For each well 0 .5μg plasmid was combined with 25 μg polyethyleneimine (PEI) in 0 .5ml serum-free medium and incubated at RT for 30 min. The medium on the cells was changed to serum-free medium and the transfection mix added. Cells were incubated for 4 h at 37 °C then the medium replaced with complete medium and the incubation continued. MeAIB uptake assays were carried out 18–24 h later using [^14^C] Me-AIB tracer.

### 2.6 Transfection of HEK 293T cells for activity assays

HEK 293T cells were seeded in 10 cm dishes. 5 μg of pShuttleCMV/CDK plasmid was mixed with 500 μl serum-free medium and 25 μg PEI and incubated at RT for 30 min. The medium on the cells was replaced with serum-free medium and the transfection mix added. Cells were incubated for 4 h at 37 °C then the medium replaced with complete medium. Cells were lysed the following day as described above and used for activity assays.

### 2.7 Kinase activity assays

GSK3β kinase activity assays have been described previously [53]. pShuttleCMV/CDK7-V5 was transfected into the HEK293T and lysates prepared as described above. Recombinant V5-tagged CDK7 was immunoprecipitated from 1 mg lysate for 2 h at 4 °C using protein G-sepharose beads that had previously been conjugated to V5 antibody at a ratio of 1 μl antibody per 20 μl slurry. Antibody-bead complexes were washed twice in lysis buffer containing an additional 0 .5M sodium chloride and then twice in CDK7 wash buffer (20 mM Tris pH 7.5, 7 .5mM MgCl_2_). Beads were resuspended to a final volume of 25 μl in CDK7 assay buffer (20 mM Tris pH 7.5, 7.5 mM MgCl_2_, 0 .2μg/μl histone H1, 10 μM non-radiolabelled ATP and 5 μCi [^32^P] ATP) and incubated shaking for 1 h at 37 °C. 15 μl of reaction volume was spotted onto p81 filter paper. The filter papers were allowed to dry and then washed 3 times in 1% phosphoric acid and then once in acetone. Filter papers were dried and [^32^P] ATP incorporation measured using a Beckman LS 6000IC scintillation counter.

pShuttleCMV/CDK9-V5 was transfected into the HEK293T and lysates prepared as described above. Recombinant V5-tagged CDK9 was immunoprecipitated from 1 mg lysate in a similar manner as for CDK7. Antibody bead complexes were washed twice in lysis buffer containing an additional 0 .5M sodium chloride and then twice in CDK9 wash buffer (50 mM Tris pH 7.4, 0.1 mM EDTA, 10 mM magnesium acetate). Beads were resuspended to a final volume of 25 μl in CDK9 assay buffer (50 mM Tris pH 7.4, 0.1 mM EDTA, 10 mM magnesium acetate, 10 mM DTT, 1 mg/ml BSA, 0 .3mM CDK9 substrate (YSPTSPSYSPTSPSYSPTSPSKKK) (MRC Reagents and Services, University of Dundee), 5 μM non-radiolabelled ATP and 5 μCi [^32^P] ATP) and incubated shaking for 1 h at 22 °C. 15 μl of reaction volume was spotted onto p81 filter papers and the filter papers washed and counted as above.

pShuttleCMV/CDK5-V5 and pShuttleCMV/p35-FLAG were co-transfected into the HEK293T and lysates prepared as described above. Recombinant V5-tagged CDK5 was immunoprecipitated from 0 .5mg lysate for 2 h at 4 °C using protein G-sepharose beads that had previously been conjugated to V5 antibody at a ratio of 1 μl antibody per 20 μl slurry. Endogenous CDK5 was immunoprecipitated from lysates generated from L6 myotubes using protein G-sepharose beads that had previously been conjugated to either V5 antibody (1 μl per 20 μl slurry) or CDK5 antibody (5 μg per 20 μl slurry). Antibody-bead complexes were washed twice in lysis buffer containing an additional 0 .5M sodium chloride then twice in CDK5 wash buffer (20 mM Tris pH 7.4, 10 mM MgCl2, 1 mM EDTA, 1 mM NaF, 0 .1mM Na_2_VO_3_). Beads were resuspended in 50 μl CDK5 assay buffer (20 mM Tris pH 7.4, 10 mM MgCl2, 1 mM EDTA, 1 mM NaF, 0 .1mM Na_2_VO_3_, 0 .2μg/μl histone H1, 0 .1mM non-radiolabelled ATP and 5 μCi [32P] ATP) and incubated shaking for 30 min at 30 °C. 25 μl of reaction was spotted onto p81 filter paper. The filter papers were dried then washed 3 times in 0.75% phosphoric acid and once in acetone before being dried and counted as above.

### 2.8 RNA extraction, cDNA synthesis and real-time PCR

RNA was extracted from cells using TriReagent according to the manufacturer's instructions. First-strand cDNA was synthesised from 1 μg of total RNA using oligo(dT)15 primers and MMLV reverse transcriptase according to the manufacturer's instructions. Real-time quantitative PCR was performed using a StepOne Plus real-time thermocycler (Applied Biosystems), SYBR Green JumpStart kit (Sigma–Aldrich) and primers targeting SNAT2, CDK5, CHOP, ATF3, ATF4, EGR1 and GAPDH (Table 1). PCR conditions were as follows: initial denaturation at 95 °C for 2 min followed by 40 cycles of 95 °C for 15 s, 55 °C for 15 s and extension at 68 °C for 30 s. The ratio of SNAT2, ATF3, ATF4, EGR1 and CHOP expression to GAPDH mRNA expression was calculated using a method described previously [46].

### 2.9 Generation of shRNA-containing lentivirus and production of stable cell lines

Stable L6 CDK5 knockdown cell lines were generated in a similar manner to that described previously [34,53] using the pLKO.1-puro vector and the oligos detailed in Table 1. The control hairpin sequence was that used previously. Stable cell lines were established using early passage cells and, once established, were only used for a maximum of five passages. Knockdown efficiency of CDK5 was assessed using western blotting and real-time PCR.

### 2.10 Statistical analyses

For multiple comparisons, statistical analysis was performed using one-way ANOVA. For individual comparisons, statistical analysis was performed using Student's *t*-test. Data analysis was performed using GraphPad Prism software and considered statistically significant at *P* < 0.05.

## 3 Results and discussion

### 3.1 Effects of GCN2, MEK/ERK and GSK3 inhibition on System A/SNAT2 activity/expression in response to amino acid deprivation

**Fig. 1:**
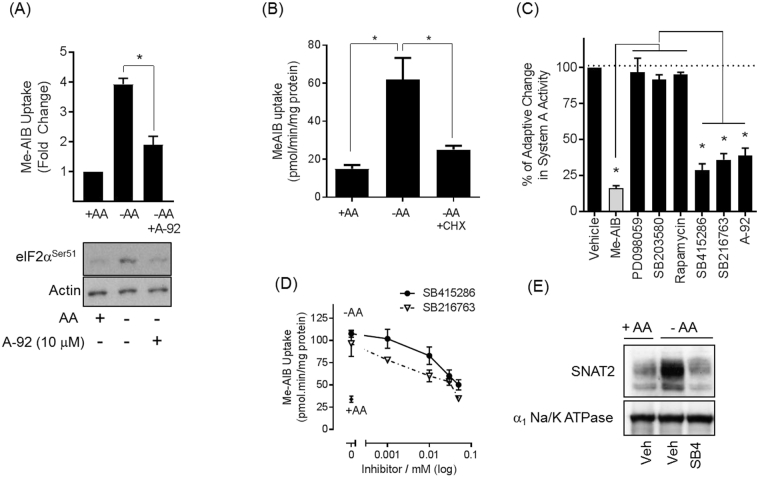
Effects of protein synthesis and kinase inhibitors on System A/SNAT2 adaptation in response to AA withdrawal. (A) L6 myotubes were deprived of amino acids (−) or not (+) for 6 h in the presence/absence of 10 μM A-92 (GCN2 inhibitor). Cells were either used to assay Me-AIB uptake (upper panel) or subjected to western blotting using the antibodies shown. (B). L6 myotubes were deprived of amino acids (−) or not (+) for 6 h in the presence/absence of 5 μg/ml cyclohexamide (protein synthesis inhibitor) then the rate of Me-AIB uptake assayed. (C) L6 myotubes were deprived of amino acids (−) or not (+) for 6 h in the presence/absence of 10 μM unlabelled Me-AIB, 50 μM PD098059 (MEK inhibitor), 10 μM SB203580 (p38 MAPK inhibitor), 100 nM rapamycin (mTORC1 inhibitor), 50 μM SB415286 or 5 μM SB216763 (GSK3 inhibitors) and 10 μM A-92 prior to analysis of Me-AIB uptake. The adaptive increase in System A transport induced by AA withdrawal in myotubes in the absence of any inhibitors was assigned a value of 100% (vehicle control). The adaptive increase in System A transport measured in the presence of Me-AIB or the different kinase inhibitors was then expressed relative to that of the vehicle control condition. (D) L6 myotubes were incubated with or without amino acids for 6 h in the presence of SB216763 or SB415286 before being assayed for Me-AIB uptake. (E) L6 myotubes were incubated for 6 h in the presence/absence of amino acids and 50 μM SB415286. Total membranes were prepared, subjected to SDS-PAGE and immunoblotted using the antibodies against SNAT2 and the alpha1 subunit of the Na,K-ATPase. Blots are representative of at least 3 independent experiments. Quantified values are presented as the mean ± SEM. *P < 0.05.

The adaptive upregulation of System A/SNAT2 transport in response to cellular amino acid insufficiency is a remarkably well preserved phenomenon [21]. Fig. 1A shows that L6 myotubes subjected to extracellular AA withdrawal for 6 h exhibit a 4-fold increase in Me-AIB uptake; a paradigm non-metabolisable System A substrate. Consistent with the idea that the adaptive response is primarily dependent on increased expression and translation of SNAT2 mRNA and that this requires activation of GCN2, the observed increase in Me-AIB uptake was significantly blunted by A-92 (a GCN2 inhibitor, [8]) and cycloheximide (a eukaryotic protein synthesis inhibitor) when they were present during the AA withdrawal period (Fig. 1A and B). Fig. 1A lower panel shows that under these circumstances, AA deprivation induces phosphorylation of eIF2α, the primary GCN2 target, and that this is suppressed by the presence of A-92.

In order to garner some insight into whether MAP kinases (MEK/ERK and p38 MAP kinases) and/or GSK3 may support the adaptive increase in System A we subsequently investigated what impact targeting these kinases with pharmacological inhibitors would have on the adaptive response. As a positive inhibitory control, we have shown previously that the System A adaptation response is critically dependent upon substrate availability/carrier occupancy [23]. In line with this, the adaptive increase in System A transport in L6 myotubes maintained in AA-free EBSS, but supplemented with 2 mM Me-AIB alone was markedly reduced (Fig. 1C). Consistent with the data presented in Fig. 1A, the presence of the GCN2 inhibitor (A-92) during the AA withdrawal period suppressed the adaptive increase in System A transport. In contrast, neither PD098059 nor SB203580 (which respectively target MEK/ERK and p38 MAP kinases) had any detectable effect upon the adaptive increase in AA-deprived myotubes. It should be stressed that the efficacy of both inhibitors, in terms of suppressing the insulin- or stress-induced activation of these kinases, was validated in parallel experiments (data not shown). Rapamycin, which inhibits the AA-regulated mTORC1/S6K1 signalling axis [45], was also without effect, whereas, both SB415286 and SB216763 (two well established but structurally distinct GSK3 inhibitors [12]) significantly blunted the adaptive increase in System A-mediated Me-AIB uptake in a dose dependent manner (Fig. 1C and D). The inhibition in transport activity caused by SB415286 was associated with a reduction in the cellular SNAT2 abundance that is normally seen in AA-deprived cells (Fig. 1E).

### 3.2 Effects of GSK3α/β gene silencing on the SNAT2 adaptive response

**Fig. 2:**
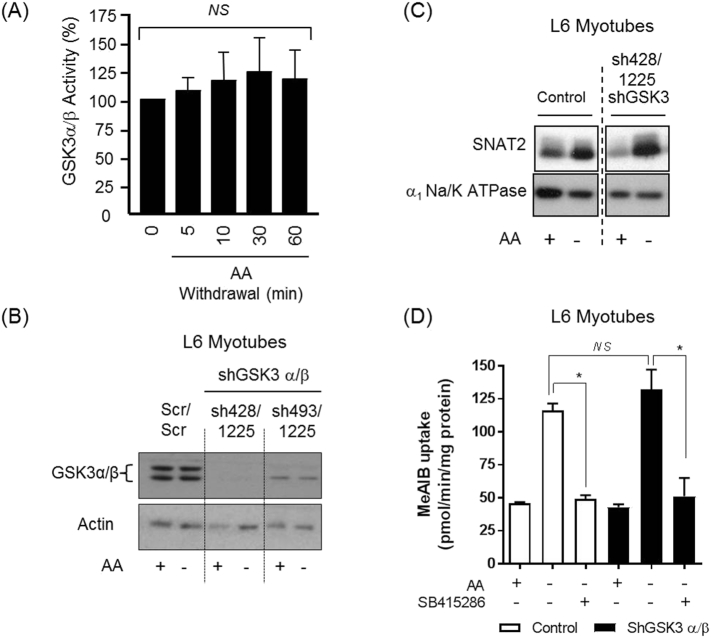
Effects of AA withdrawal on GSK3 α/β activity and effects of GSK3 α/β silencing on System A/SNAT2 adaptation in L6 myotubes. L6 myotubes were acutely deprived of amino acids (AA-) for the times indicated prior to assaying total GSK3 (α/β) activity (A). Control and GSK3 silenced cells were incubated in the presence/absence of amino acids for 6 h and used to prepare total cell lysates (B), total membranes (C) or assay Me-AIB uptake (D). Blots showing GSK3 and actin (loading control) are representative of at least 3 individual experiments. Quantified values are presented as the mean ± S.E.M. *P < 0.05. *NS*, not significant.

**Fig. 3:**
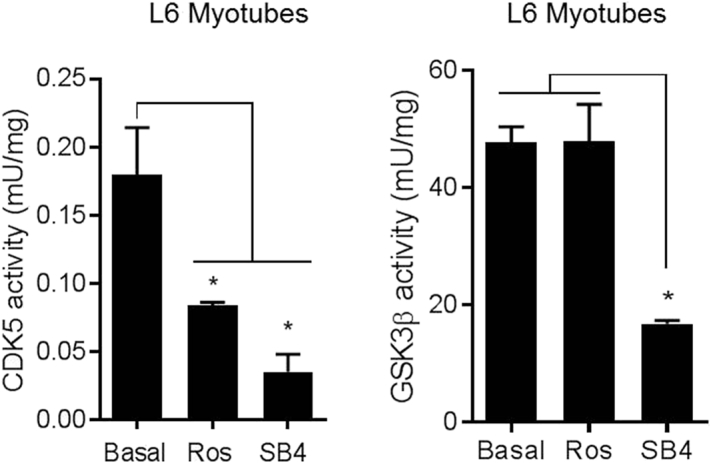
Effects of roscovitine and SB415286 on CDK5 and GSK3β activity in L6 myotubes. Wild-type L6 myotubes were incubated in EBSS containing amino acid mix at plasma physiological concentrations for 6 h in the presence/absence of 50 μM SB415286 or 30 μM roscovitine as indicated. Cell lysates were prepared and either 500 μg or 100 μg subjected to immunoprecipitation with CDK5 or GSKβ antibodies respectively. Kinase activity assays were carried out using histone H1 or phospho-GS peptide respectively as substrates. Activities are presented as the mean ± S.E.M (*n* = 3).

The data presented in Fig. 1C–E implicated GSK3 in the adaptive regulation of SNAT2. To explore the involvement of this kinase further we assessed whether its activity was modulated in AA-deprived cells. Fig. 2A shows that there was no discernible change in GSK3 activity in L6 myotubes when these were AA-deprived for up to 1 h. GSK3 is constitutively active in most mammalian cells and it is plausible that whilst AA availability has no direct impact on the kinase that its activity is an integral part of the AAR. To test this proposition we utilised lentivirus-based shRNA technology to generate L6 myotubes exhibiting stable knockdown of both GSK3α and GSK3β [53]. In two separate clonal L6 cell lines (sh428/1225 and sh493/1225) we were able to get substantial depletion (>90%) of both GSK3 isoforms (Fig. 2B). Surprisingly, however, despite this significant depletion in GSK3 expression, subjecting GSK3-silenced cells to AA deprivation for 6 h did not abate the adaptive increase in SNAT2 protein abundance (Fig. 2C) nor the associated increase in functional transport activity that remained sensitive to inhibition by SB415286 (Fig. 2D). These latter observations imply that it is highly unlikely that GSK3 is a component of the AAR and that the effects of SB415286 (and SB26763) most likely represent an off-target effect. Consistent with this idea, there is evidence that SB415286 and SB26763 can also inhibit members of the cyclin-dependent kinase (CDK) family *in vitro*, which are well known targets of the olomoucine derivative, roscovitine [4]. To test the possibility of an off-target effect we assayed the sensitivity of CDK5 (as a representative member of the CDK family) and GSK3β to SB415286 and roscovitine. Fig. 3 shows that immunoprecipitable CDK5 activity from L6 myotubes was reduced when assayed in the presence of both roscovitine and SB415286 using an *in vitro*-based kinase assay. In contrast, roscovitine had no detectable effect upon GSK3β activity.

### 3.3 Effects of CDK inhibitors on the System A/SNAT2 adaptive response

**Fig. 4:**
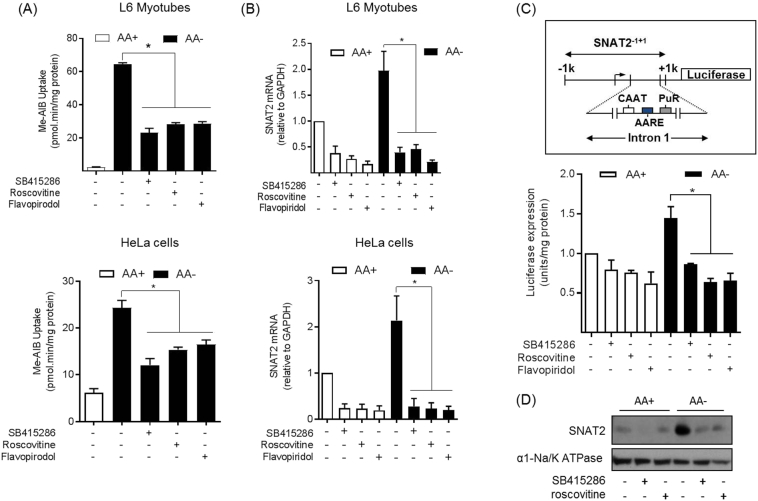
Effects of SB415286, roscovitine and flavopiridol on System A/SNAT2 adaptation in response to AA insufficiency in L6 myotubes and HeLa cells. (A) Wildtype L6 myotubes and HeLa cells were deprived of amino acids (−) or not (+) for 6 h in the presence/absence of 50 μM SB415286, 30 μM roscovitine or 500 nM flavopiridol as indicated. Following incubation the rate of MeAIB uptake was assayed. (B) Wildtype L6 myotubes and HeLa cells were deprived of amino acids (−) or not (+) for 6 h in the presence of 50 μM SB415286, 30 μM roscovitine or 500 nM flavopiridol as indicated. RNA was isolated, cDNA synthesised and expression of SNAT2 mRNA was determined by quantitative PCR analysis. (C) L6 myotubes stably expressing a pcDNA3 luciferase reporter construct were deprived of amino acids (−) or not (+) for 8 h in the presence/absence of 50 μM SB415286, 30 μM roscovitine or 500 nM flavopiridol as indicated and luciferase expression subsequently assayed. Quantified values in all panels are presented as the mean ± S.E.M. (*n* = 3). (D) Wild-type L6 cells were deprived of amino acids (−) or not (+) for 6 h in the presence/absence of 50 μM SB415286 or 30 μM roscovitine then total membranes prepared and subjected to SDS-PAGE followed by immunoblotting for SNAT2 and the α1 subunit of Na/K ATPase as a loading control. Blots are representative of at least three independent experiments. Asterisk represents a significant difference between the indicated bars (P < 0.05).

Having established that SB416286 could inhibit a CDK family member *in vitro* we subsequently explored what effect roscovitine and flavopiridol (another pan CDK-inhibitor) may have upon the System A/SNAT2 adaptive response in L6 muscle cells. Fig. 4A and B (upper panels) shows that both roscovitine and flavopiridol were as effective as SB415286 in reducing the adaptive increase in Me-AIB uptake in L6 myotubes that is induced by a 4 h period of AA deprivation and that this loss in functional transport activity was also associated with a marked reduction in both basal and SNAT2 mRNA abundance. The observed sensitivity of the AAR to roscovitine and flavopiridol was not restricted to L6 myotubes as the effect of both CDK inhibitors on the AAR was also seen in the human cervical cancer cell line, HeLa (Fig. 4A and B; lower panels). To further substantiate that CDK inhibition was associated with a reduction in the AAR invoking an increase in SNAT2 gene transcription we utilised L6 cells that had been stably transfected with a pcDNA3 luciferase reporter construct, driven by the SNAT2 promoter that included the first intronic element containing the tripartite AAR domain [23]. Fig. 4C shows AA deprivation enhances luciferase expression, but that this was absent when cells were exposed to SB415286 or inhibitors targeting the CDK family during the AA withdrawal period. Furthermore, consistent with the data shown in Fig. 4A, the increase in SNAT2 protein induced by AA deprivation in L6 myotubes was also repressed by roscovitine (Fig. 4D).

### 3.4 CDK inhibitors suppress expression of ATF4, CHOP and ATF3

**Fig. 5:**
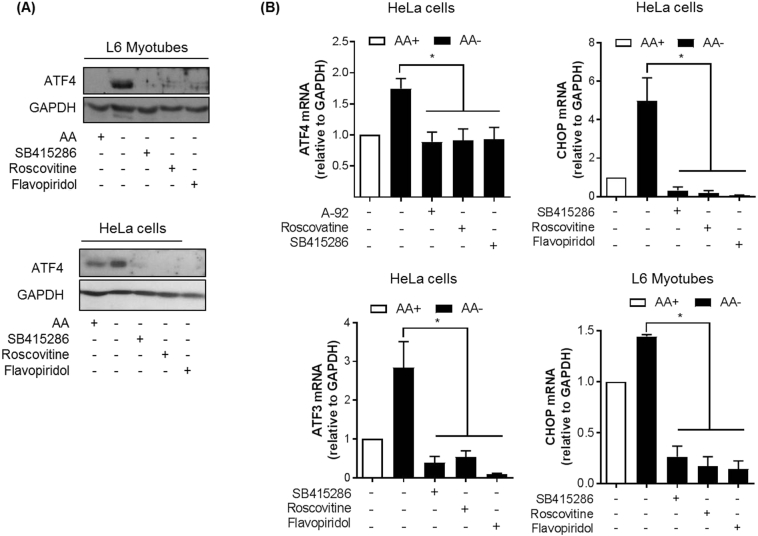
Effects of SB415286, roscovitine and flavopiridol on ATF4 and ATF4-regulated genes in L6 myotubes and HeLa cells during AA insufficiency. (A) L6 myotubes and HeLa cells were deprived of amino acids (−) or not (+) for 6 h in the presence/absence of 50 μM SB415286, 30 μM roscovitine or 500 nM flavopiridol then the cells lysed and subjected to SDS-PAGE followed by immunoblotting with the indicated antibodies. Blots are representative of at least 3 independent experiments. (B) L6 myotubes or HeLa cells were deprived of amino acids (−) or not (+) for 6 h then RNA prepared from the cells, cDNA synthesised and the expression of the indicated gene transcripts determined by quantitative PCR analysis. Data is presented as the mean ± S.E.M. (n = 3).

The adaptive increase in SNAT2 gene expression is, in part, dependent upon ATF4 [27]; a transcription factor whose expression, like that of the SNAT2 gene, is upregulated in response to AA deficiency and, which, in addition to SNAT2, supports the upregulation of genes such as CHOP and ATF3 that form as part of the AAR. Given that pan CDK inhibitors blocked SNAT2/System A adaptation and some CDKs have important roles in transcriptional control [40] we subsequently assessed what effect these inhibitors have on ATF4 and some of its transcriptional targets that are upregulated by the AAR. In line with previous studies, Fig. 5A shows that L6 myotubes or HeLa cells subjected to a 6 h period of AA deprivation exhibit a robust increase in ATF4 protein abundance, which, strikingly, was not observed when cells were AA-deprived in the presence of SB415286, roscovitine or flavopiridol. Consistent with this latter observation, the expression of ATF4, CHOP and ATF3, whose gene expression is also induced by AA withdrawal, was suppressed significantly in cells exposed to the inhibitors used (Fig. 5B).

### 3.5 Effects of expressing dominant negative forms of CDK5, CDK7 and CDK9 on the System A adaptation response

**Fig. 6:**
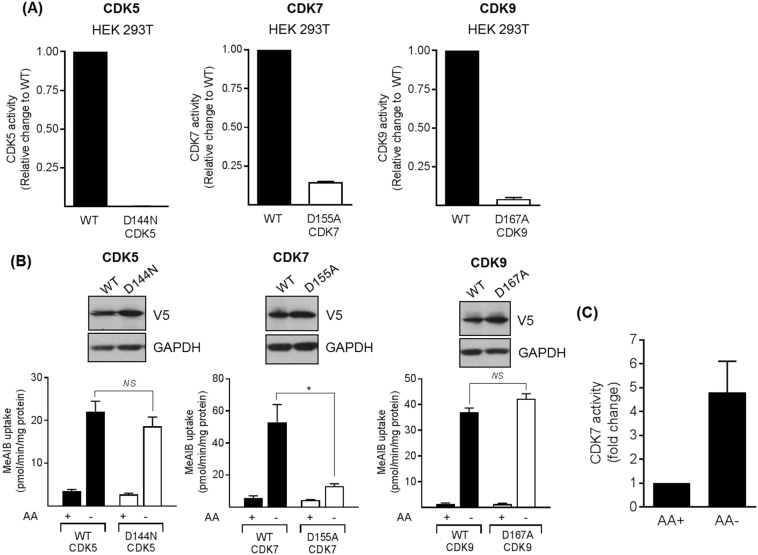
Effects of overexpressing wild-type and dominant interfering mutants of CDK5, CDK7 and CDK9 upon System A adaptation and effects on AA withdrawal (AA-) on CDK7 activity in HEK293T cells. (A) HEK293T cells were transfected with 5 μg of the following vectors; pShuttleCMV/CDK5-WT, pShuttleCMV/CDK5-D144N, pShuttleCMV/p35-FLAG, pShuttleCMV/CDK7-WT, pShuttleCMV/CDK7-D155A, pShuttleCMV/CDK9-WT and pShuttleCMV/CDK9-D167A as indicated. Cell lysates were prepared and the over-expressed CDKs immunoprecipitated from either 500 μg (CDK5) or 1 mg (CDK7 and CDK9) lysate using antibody targeted to the V5 tag. Activity assays for CDK5, CDK7 and CDK9 were carried out. Data is expressed as a relative change to the wild type kinase activity. (B) Cells were transduced with adenovirus expressing either V5 tagged WT or DN forms of CDK5 (D144N), CDK7 (D155A) and CDK9 (D167A). 24 h post transfection cells were incubated in the presence/absence of amino acids for 6 h prior to assaying Me-AIB uptake or (C) analysis of CDK7 activity in V5 immunoprecipitates. Data in all cases is presented as the mean ± S.E.M. (*n* = 3). Blots are representative of at least three independent experiments. NS, not significant

**Fig. 7:**
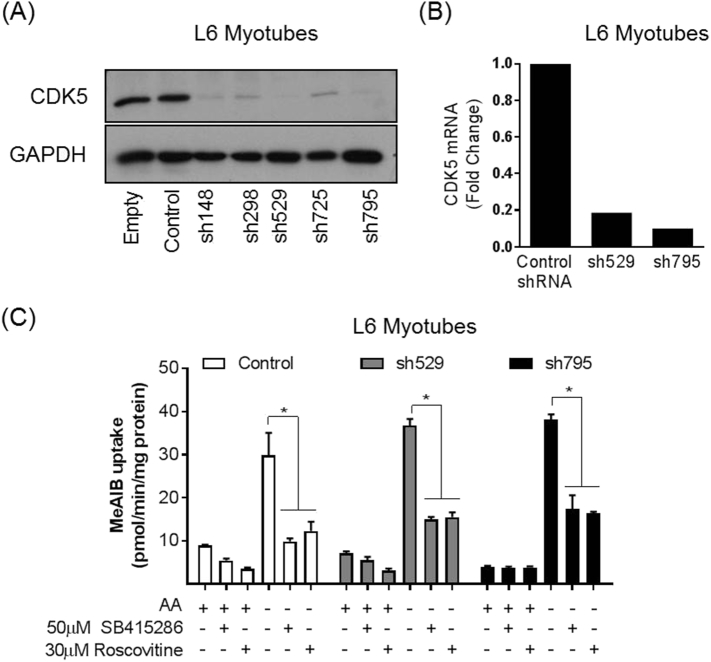
CDK5 shRNA silencing in L6 myotubes does not affect System A adaptation in response to AA insufficiency L6 myotubes were stably transduced with lentiviral vectors expressing different shRNAs targeting murine CDK5. Cells were lysed, separated by SDS-PAGE and immunoblotted using antibodies targeting CDK5 and GPADH (A) or RNA isolated, cDNA synthesised and CDK5 mRNA assayed by quantitative PCR (B). Blots and bar graphs are representative of three individual experiments. Stably transduced L6 myotubes were amino acids deprived for 6 h in the presence/absence of 50 μM SB415286 or 30 μM roscovitine and MeAIB uptake assayed (C). Values are mean ± SEM (n = 3).

Roscovitine and flavopiridol are pan-CDK inhibitors and consequently do not allow discrimination of which CDK isoform(s) may participate in the AAR. However, in addition to their critical role in the cell cycle, it is widely accepted that some members of the CDK family have other key cellular functions [13,15]. For example, CDK5 has little involvement in the cell cycle but has been implicated in the regulation of cytoskeletal elements, synaptic function and membrane transport events (see review [13]), whereas CDK7 and CDK9 belong to the transcriptional sub-family of CDKs [15]. Since the inhibitory effects of roscovitine and flavopiridol on the AAR are evident in terminally differentiated L6 myotubes (in which there is minimal cell cycle activity) we reasoned that CDK5 and those influencing transcriptional control (*i.e.* CDK7 and CDK9) may be good candidates for further investigation. To test their possible involvement we explored what impact cellular expression of V5-tagged dominant–interfering forms of CDK5 (D144N) [14], CDK7 (D155A) [49] and CDK9 (D167A) [16] may have on the adaptive response. Fig. 6A shows that unlike the respective wild-type kinases, the dominant-inhibitory forms of CDK5, CDK7 and CDK9 all lack catalytic activity when assessed in immunoprecipitates of the respective V5-tagged kinases expressed in HEK293T cells. Subsequent cellular expression of wild type CDK5 and CDK9 or their dominant-interfering forms had no detectable impact on the adaptive increase in Me-AIB uptake when assayed in cells that had been AA deprived for 6 h (Fig. 6B). The lack of any CDK5 involvement in the AAR was further validated using L6 myotubes in which CDK5 had been stably silenced by >90% using a shRNA-lentiviral based strategy (Fig. 7). Despite the substantial reduction in CDK5 expression, the System A adaptive response (assessed using Me-AIB uptake as a readout) was unaffected when assayed in two separate L6 CDK5 knock-down clones and, as such, the AAR remains sensitive to SB415286 and roscovitine in the CDK5-silenced cell lines (Fig. 7). However, unlike cells expressing CDK5-D144N or CDK9-D167A, the expression of CDK7-D155A in HEK293T cells resulted in significant blunting of the adaptive response supporting its potential involvement in the AAR (Fig. 6B). This latter proposition was further supported by our finding that CDK7 activity was elevated by nearly 4–5-fold when HEK293T cells were AA deprived for 6 h (Fig. 6C).

### 3.6 Targeted inhibition of CDK7 blunts the System A/SNAT2 adaptive response

**Fig. 8:**
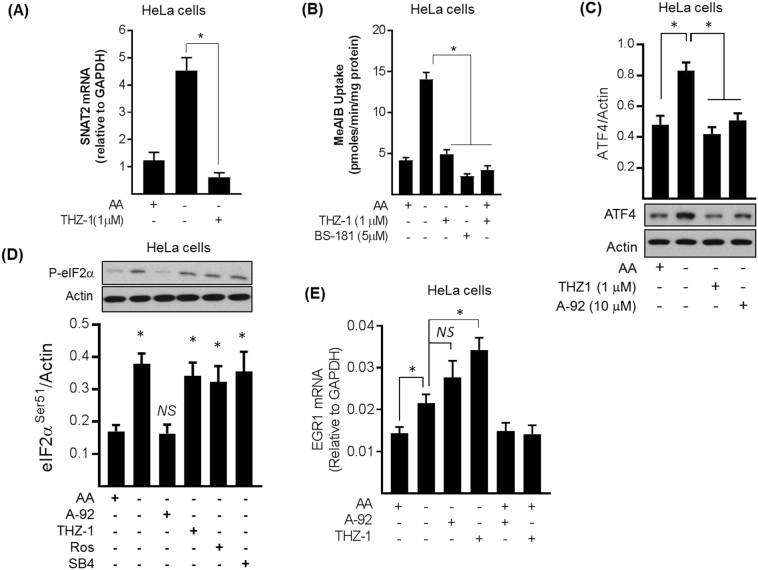
Effects of THZ-1, BS-181 and A-92 on SNAT2 mRNA expression, System A adaptation and ATF4 protein abundance in response to AA withdrawal in HeLa cells HeLa cells were deprived of amino acids (−) or not (+) for 6 h in the presence or absence of 1 μM THZ-1 (A, B, C), 5 μM BS-181 (B) or 10 μM A-92 (C) as indicated. Following cell treatment, expression of SNAT2 mRNA was determined by quantitative PCR analysis of isolated RNA (A). Alternatively, Me-AIB uptake was measured following the incubation period (B). ATF4 protein abundance was determined by subjecting resulting cell lysates to SDS-PAGE and then immunoblotting using anti-ATF4 and anti-actin (used as loading control) antibodies as shown (C). Quantified values are presented as the mean ± S.E.M. (n = 3). *, *P* < 0.05. *NS*, not significant.

To further substantiate involvement of CDK7 in the AAR, we investigated the effects of THZ-1 and BS-181 (two highly selective CDK7 inhibitors with IC50 values of 3.2 and 21 nM, respectively [1,29]). In line with the data presented in Fig. 1, subjecting HeLa cells to a 6 h period of AA deprivation induced a significant increase in both SNAT2 mRNA abundance and Me-AIB uptake, neither of which was apparent in cells treated with THZ-1 during the AA withdrawal period (Fig. 8A, B). Likewise, the adaptive increase in System A transport was also repressed by the structurally unrelated CDK7 inhibitor, BS-181 (Fig. 8B). As indicated earlier, pan CDK inhibitors repress the increase in ATF4. To confirm that such repression is indeed mediated by CDK7, analysis of ATF4 protein in HeLa cells that had been AA-deprived in the absence and presence of THZ-1 revealed that the increase in ATF4 was blocked by CDK7 inhibition and, as anticipated, also under circumstances when GCN2 was inhibited using A-92 (Fig. 8C). It is possible that the repression we see in ATF4 protein in AA deprived cells in the presence of pan CDK inhibitors (SB415286, roscovitine and flavopiridol, Fig. 5A) and THZ-1 (Fig. 8C) may reflect an off-target effect on GCN2. To test this possibility the effects of AA withdrawal on eIF2α phosphorylation (taken as a readout of GCN2 activity) was determined in the presence of A-92, roscovitine, SB415286 and THZ-1. Fig. 8D shows that AA deprivation induced ~2-fold increase in eIF2α phosphorylation, which was unaffected by the presence of roscovitine, SB415286 or THZ-1 but lost in cells exposed to the GCN2 inhibitor, A-92. Collectively, the data presented in Fig. 8A–D indicate that the adaptive increase in SNAT2/System A expression/activity is reliant not only upon the GCN2/ATF4 pathway, but that CDK7 is likely to play an important role in the transcriptional upregulation of ATF4 target genes. To further test the specificity and involvement of CDK7 in the AAR we assessed the expression of the early growth response gene (EGR1) whose activation in response to AA deprivation is known to be independent of the GCN2-ATF4 pathway, but dependent on MEK-ERK signalling [51]. Fig. 8E shows that we observed a modest, but significant increase in EGR1 expression in AA deprived HeLa cells and that, as anticipated, this was not inhibited by the presence of A-92, which, in fact, had a modest stimulatory effect on EGR1 expression. It is noteworthy that previous work utilising HepG2 cells stably expressing shRNA against GCN2 also found that, relative to parental cells or those expressing a control shRNA sequence, the AAR induced a much greater level of EGR1 expression [51]. Likewise, whilst THZ-1 had no inhibitory effect on EGR1 expression, it too augmented expression of the EGR1 gene. Since we postulate that CDK7 activation by AA withdrawal is downstream of GCN2, these observations might indicate that whilst CDK7 can promote expression of SNAT2 during the AAR it may also exert a repressive effect on non-ATF4 regulated stress genes such as EGR1, which we can alleviate by inhibition with THZ-1. The notion that some CDKs may function as both positive and negative regulators of transcription is not unprecedented [40].

### 3.7 Amino acid deprivation induces CDK7-mediated phosphorylation of the CTD of RNA polymerase II

**Fig. 9:**
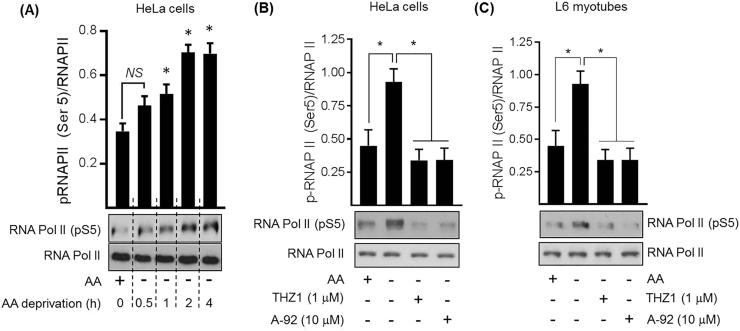
Effects of AA withdrawal, THZ-1 and A-92 on Pol II Ser5 phosphorylation in HeLa cells. HeLa cells were deprived of amino acids (−) for the time periods shown (A). In addition, HeLa cells (B) or L6 myotubes (C) were deprived of amino acids (−) or not (+) for 6 h in the presence or absence of 1 μM THZ1 and 10 μM A-92 as indicated (B, C). Following indicated treatments, resulting cell lysates were subjected to SDS-PAGE followed by immunoblotting using the antibodies shown. Quantified values are the mean ± S.E.M. from three independent experiments. *P < 0.05 between indicated bar values. *NS*, not significant.

CDK7 forms a heterotrimeric complex with cyclin H and MAT1 and functions not only as a CDK-activating kinase (CAK), but as a crucial component of the general transcription factor TFIIH. A key physiological target for the TFIIH/CDK7 complex is the C-terminal domain (CTD) of the large subunit of RNA Polymerase II (RNAPII), which, in eukaryotic cells, contains 52 repeats of a heptapeptide (Y_1_S_2_P_3_T_4_S_5_P_6_S_7_) sequence [56]. CDK7 phosphorylates Ser5 in these heptads within the CTD of RNA polymerase II (RNAPII) and this is considered to facilitate RNAPII promoter escape and transitioning to the elongation step of gene transcription [41]. Consistent with the increase in CDK7 activity that we see in AA starved cells (Fig. 6C), AA deprivation induced a time-dependent increase in Ser5-phosphorylation of the Pol II CTD in HeLa cells (Fig. 9A). This phosphorylation was abolished by cell treatment with THZ-1; a selective and potent CDK7 inhibitor, but intriguingly also by the GCN2 inhibitor, A-92, in both HeLa cells and L6 myotubes (Fig. 9B and C). We currently do not know how CDK7 activation is mechanistically linked to the AA withdrawal stimulus, but the observation that A-92 suppresses phosphorylation of a CDK7 target implies that GCN2 activation in AA-deprived cells is somehow involved and that this event is upstream of CDK7. Since GCN2 activation promotes synthesis of basic-region leucine zipper DNA binding proteins such as ATF4, and expression of the latter in AA deprived cells is impaired by THZ-1 (Fig. 8C), our findings would suggest that CDK7 may potentially link GCN2 to control of ATF4 gene expression, which then subsequently may help to regulate expression of nutrient transporters such as SNAT2 as part of the AAR.

### 3.8 Expression of a doxycycline-inducible drug-resistant CDK7 mitigates THZ-1 inhibition of the System A adaptive response

**Fig. 10:**
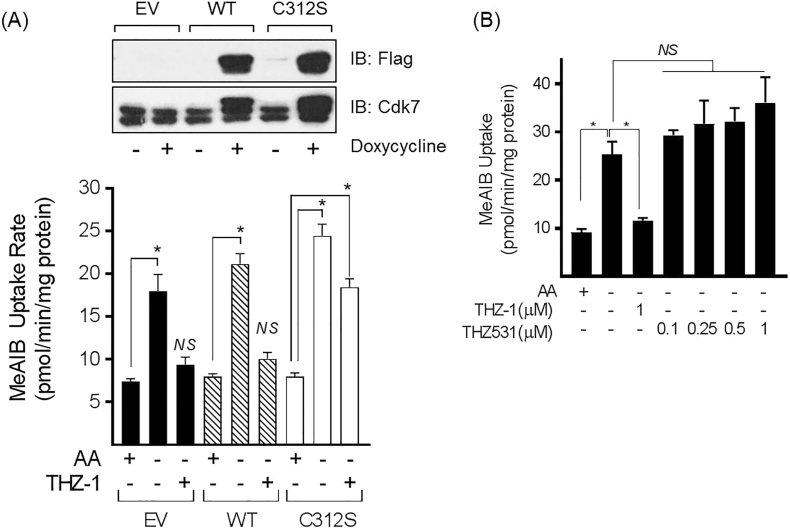
Effects of expressing a doxycycline-inducible drug-resistant CDK7 on System A adaptation in response to AA withdrawal. HeLa S3 cells stably expressing doxycycline-inducible FLAG-WT CDK7 or FLAG-C132S CDK7 constructs (or empty vector (EV)) were treated with (+) or without (−) doxycycline (2 μg/ml) for 24 h. Resulting cell lysates were subjected to SDS-PAGE and immunoblotted using the antibodies indicated (A). Alternatively, doxycycline treated cells were deprived of amino acids (−) or not (+) for 6 h in the presence or absence of 1 μM THZ-1 prior to measurement of Me-AIB uptake rates (A). In addition, wild type HeLa cells were starved of amino acids (−) or not (+) for 6 h in the presence or absence of 1 μM THZ-1 and the indicated concentrations of THZ531 (B). Quantified values are presented as the mean ± S.E.M. (n = 3). *, *P* < 0.05; *NS*, not significant relative to corresponding amino acid containing control unless otherwise indicated.

Recent studies have indicated that the exceptional sensitivity of CDK7 to THZ-1 is due to the covalent interaction that the inhibitor forms with Cys312, which traverses the ATP cleft in the kinase domain resulting in its irreversible inhibition. Strikingly, replacing Cy312 with a less nucleophilic amino acid (Ser), ablates the interaction with the inhibitor and renders the kinase resistant to THZ-1 [29]. To test if the observed loss in the adaptive upregulation of System A/SNAT2 that we see in response to THZ-1 could be prevented we utilised HeLa S3 cells expressing doxycycline-inducible Flag-tagged versions of the wild type and CDK7-C312S mutant [29]. Using Flag antibodies, Fig. 10A (upper panel) shows that 24 h of doxycycline treatment induces expression of the wild-type (WT) and CDK7-C312S mutant kinases, whereas cells transfected with the empty vector (EV, control) do not. The C-terminal directed anti-CDK7 antibody can give rise to two immunoreactive bands as previously noted [28], but identifies an additional band in doxycycline-treated cells that correspond to the exogenously expressed FLAG-tagged protein. Cells expressing the EV, WT and C312S vectors were subsequently either maintained in buffer containing or lacking the physiological AA mix for 6 h in the absence and presence of THZ-1 prior to analysis of Me-AIB uptake. Fig. 10A (lower panel) shows that HeLa S3 cells expressing the EV or WT CDK7 exhibit the adaptive increase in System A transport upon AA deprivation, which, as anticipated, was attenuated in cells treated with THZ-1. However, cells expressing the drug resistant CDK7-C312S mutant retain a significant adaptive response in the presence of THZ-1 despite the inhibitory effect this drug has upon the endogenously expressed CDK7 protein. Whilst THZ-1 is a potent CDK7 inhibitor, at high concentrations it can potentially target CDK12 [29]. To test whether the effects of THZ-1 were a consequence of CDK12 inhibition, we utilised THZ531; a highly selective covalent inhibitor of CDK12 and CDK13 (IC_50_ 158 nM and 69 nM, respectively) that has very weak action on CDK7 (IC_50_ 8.5 μM) [57]. Fig. 10B shows that unlike THZ-1, THZ531 had no impact on the adaptive increase in Me-AIB uptake when HeLa cells were subject to AA withdrawal excluding any involvement of CDK12 or CDK13 in the AAR regulating System A.

## 4 Conclusions

Like many cell stresses (*e.g.* UV radiation, heat shock, osmotic stress), AA insufficiency is known to impact negatively upon cell growth and survival and modulates signalling pathways that allow adaptation to environmental cues affecting normal cellular function. The GCN2-ATF4 pathway is one component of the ISR that enables cells to deal with nutrient-induced stress. Cellular restriction or deprivation of AAs results in GCN2 activation and this induces phosphorylation of eIF2α leading, on the one hand, to a global suppression of mRNA translation and, on the other, translation and transcription of ATF4-regulated genes that facilitate adaptation and recovery of cells to AA deficiency. In addition, GCN2 has also been implicated in cell cycle arrest *via* its ability to selectively upregulate, through phosphorylation of eIF2α, the translation of a cell cycle inhibitor, p21^Cip1^, which subsequently restrains G_1_/S transition [31]. In this regard, GCN2 functions as a novel checkpoint suppressing cellular proliferation under circumstances of nutrient insufficiency and, as such, this allows cells to survive nutritionally unfavourable conditions. These latter observations underscore the inter-dependent relationship between components of the ISR and cell cycle checkpoint controls, which have previously also been highlighted for other stress-activated kinases such as SAPK/JNK and p38 MAP kinases [44].

The genomic-driven increase in SNAT2/System A transport activity seen in cells upon extracellular AA limitation represents a well-established component of the ISR [17,22,32] that is crucially dependent upon activation of the GCN2/ATF4 pathway. Our initial observations suggested that GSK3 may be a component of this ISR; a possibility supported by a study in cells of neuronal lineage which presented evidence that expression of CHOP, a transcription factor involved in the ISR, was regulated in a GSK3-dependent manner [38]. However, it is noteworthy that the proposed involvement of GSK3 in this study was based on using inhibitors of the indirubin and paullone class that are also known to target members of the CDK family [3], therefore further validation that GSK3 is involved in the ER stress response triggering CHOP expression seems warranted. Indeed, a very recent study in vascular smooth muscle cells demonstrated that whilst CHOP expression in response to saturated fatty acid-induced ER stress was repressed by kenpaullone (a GSK3/CDK inhibitor), this inhibitor-sensitive induction of CHOP was, in fact, mediated by CDK9 [52], which, like CDK7, also participates in the regulatory control of RNA transcripts generated by RNA polII [48].

Significantly, the current work identifies CDK7 as a novel component of the ISR mediating the upregulation of SNAT2 expression/function in cells subject to AA starvation. Our studies reveal, for the first time, that AA withdrawal promotes an increase in CDK7 activity and that this is associated with enhanced Ser 5 phosphorylation of the CTD of RNAPII that may help promote greater transcription of target genes involved in stress adaptation. It is currently unclear whether the SNAT2 gene itself is a downstream target of the increased CDK7 activity or if its upregulation is a secondary consequence of the kinase targeting expression of the ATF4 gene. Since translation of ATF4 mRNA transcripts would be preferentially enhanced under circumstances when eIF2α is phosphorylated this would be expected to subsequently promote expression of ATF4-regulated genes such as CHOP, ATF3 and SNAT2. Precisely how CDK7 is activated in response to AA deprivation is unclear but, since CDK7 mediated phosphorylation of the CTD of RNAPII is sensitive to A-92, our findings would imply that GCN2 activation is mechanistically linked to the downstream activation of CDK7. We accept that global changes in the cellular level of Ser 5 phosphorylation within the CTD of RNAPII may not necessarily correlate with changes taking place at the regulatory region(s) of target genes where the polymerase acts and, consequently, there is value in future studies trying and establish how genetic or pharmacological modulation of CDK7 might affect pSer5 RNAPII phosphorylation within the promoter regions of target genes. We also currently do not know whether the GCN2-CDK7 link is direct or indirect, but there is growing evidence in the literature that substrates other than eIF2α can mediate the stress-induced effects of GCN2 in both yeast and mammalian cells [39]. Whether GCN2 physically interacts with CDK7 or can influence phosphorylation of the latter on either one of its two T-loop residues (Ser164 or Thr170) that are crucial for the kinase to form a stable complex with cyclin H and MAT1 *in vivo* and for its CTD kinase activity [30] remain interesting, but open questions for future investigation.

## Conflict of interests

None of the authors have any conflict of interests to declare in relation to the current study.

## Author contributions

CS, CL, RH, EC and TH were involved in performing the experiments and data curation; CS, PT, HSH were involved in conceptualization and formal analysis. HSH wrote the original draft; CS, CL, PMT were involved in review and editing of the manuscript. PMT and HSH were involved in funding acquisition, project direction and administration.

## Transparency document

Transparency document. Image 1
